# MiR-150 regulates human keratinocyte proliferation in hypoxic conditions through targeting HIF-1α and VEGFA: Implications for psoriasis treatment

**DOI:** 10.1371/journal.pone.0175459

**Published:** 2017-04-11

**Authors:** Yongjian Li, Juan Su, Fangfang Li, Xiang Chen, Guiying Zhang

**Affiliations:** 1 Department of Dermatology, Second Affiliated Hospital of Nanhua University, Hengyang, Hunan, China; 2 Hunan Key Laboratory of Skin Cancer and Psoriasis, Department of Dermatology, XiangYa Hospital, Central South University, Changsha, Hunan, China; 3 Department of Dermatology, XiangYa Hospital, Central South University, Changsha, Hunan, China; 4 Department of Dermatology, The Second Xiangya Hospital, Central South University, Changsha, Hunan, China; University of Catanzaro, ITALY

## Abstract

Psoriasis is a common and chronic autoimmune skin disease which affects 2 to 3% of the world population. Abnormal proliferation of human keratinocytes is an important feature of psoriasis, along with local hypoxia and vascular abnormal growth. To leverage recent molecular findings into the personalized treatment of psoriasis, we need a strategy that integrates clinical stratification with molecular phenotyping. MicroRNAs (miRNAs) are a large family of small non-coding RNA which regulates diverse biological process, including cell proliferation, by modulating gene expression at the posttranscriptional level. In the present study, we indicated that miR-150 specifically down-regulated expressed in psoriatic skin lesions, and could inhibit HaCaT cells and primary adult human keratinocytes (HKCs)’ proliferation in either normal or hypoxia conditions; by direct targeting, miR-150 could also regulate the expression of hypoxia-inducible factor-1α (HIF-1α) and vascular endothelial growth factor A (VEGFA). In addition, we found that HIF-1α and VEGFA were highly expressed in the lesional psoriatic skin compared with the non-lesional psoriatic skin, and negatively correlated with miR-150 expression. Taken together, we indicated miR-150 regulates human keratinocytes’ proliferation in hypoxic conditions through targeting HIF-1α and VEGFA in psoriasis for the first time, and provide diagnostic markers and a novel target for psoriasis treatment.

## Introduction

Psoriasis is a common chronic inflammatory disease of skin affecting 2 to 3% of the world population. Its symptoms begin as red, scaly patches on the scalp, elbows, and knees that is related to systemic inflammation and comorbidities, such as psoriatic arthritis, diabetes, cardiovascular disease, and depression [1–6]. Abnormal proliferation of human keratinocytes is an important feature of psoriasis, along with local hypoxia and vascular abnormal growth [7, 8]. It is likely that exaggerated keratinocytes proliferation is closely related to disease expression. Especially, keratinocytes may be crucial in initiating, sustaining, and amplifying the inflammatory responses by expressing molecules involved in T-cell recruitment, retention, and activation [9]. Keratinocytes are also a relevant source of growth factors for angiogenesis [7]. The precise mechanism of exaggerated proliferation is not yet fully revealed. Recent research demonstrated that there were various factors participated in pathological progress of psoriasis, such as hypoxia [10–12].

MicroRNAs (miRNAs), a large family of highly conserved small non-coding RNA, regulates diverse biological process by modulating gene expression at the posttranscriptional level [13]. Several studies have found that miRNAs play a crucial role in regulating differentiation, proliferation, cell cycle and apoptosis [13–15]. Moreover, miRNAs acts as mediators of post-transcriptional gene silencing in both pathogenic and pathological aspects of autoimmune disorders according to the last decade researches [16]. MiRNAs have been shown to play pivotal roles in diverse developmental and cellular processes implicated in a variety of many autoimmune diseases including psoriasis [17, 18]. In previous studies, miRNAs abnormal expression was frequently reported in psoriasis [19], including miR-99a, miR-150, miR-423 and miR-197 [20]. In the present study, we determined the expression level of miR-150 in lesional psoriatic skin tissues compared with non-lesional psoriatic skin tissues, and validated the detailed role of miR-150 in human keratinocytes’ proliferation.

Hypoxic induction of hypoxia-inducible factor-1α (HIF-1α) protein in psoriatic skin has been confirmed [10]. In hypoxia condition, accelerated angiogenesis comes along with the exaggerated proliferation [10, 21]. In addition, vascular endothelial growth factor A (VEGFA), a pro-angiogenetic cytokine over-expressed in psoriatic skin, was reported to promote micrangiopathic modifications in psoriatic plaque [22]. Recent studies indicated that both HIF-1α and VEGFA could be regulated by miRNAs in many kinds of cancers [23–26]. Under hypoxia during liver regeneration, inhibition of miR-150 significantly elevated the expression of VEGFA at 48 h after transfection, HIF-1α protein expression increased to its highest level at 24 h following a 70% partial hepatectomy [27]. These inspired us to presume that miR-150 might regulate HIF-1α and VEGFA to modulate human keratinocytes proliferation in psoriasis.

In this study, we hypothesized that the disordered expression of miR-150 in the keratinocytes may correlate closely with psoriasis process. To validate this hypothesis, a series functional and mechanistic analysis was performed to investigate the role of miR-150 in HaCaT cell and HKCs proliferation, under either normal condition or hypoxic condition. Given that HIF-1α and VEGFA are closely related to cell proliferation and subsequently accelerated angiogenesis under hypoxia, we further investigate whether miR-150 was able to regulate HIF-1α and VEGFA to modulate the cell proliferation of HaCaT cells and HKCs. Taken together, we indicated miR-150 regulates human keratinocytes’ proliferation in hypoxic conditions through targeting HIF-1α and VEGFA in psoriasis for the first time, and provide diagnostic markers and a potential novel target for psoriasis treatment.

## Materials and methods

### Clinic specimens, cell lines and transfection

The study was conducted in accordance with the Declaration of Helsinki, and the protocol was approved by the Ethic Committee of The Second Affiliated Hospital of Nanhua University. 70 psoriasis patients diagnosed by dermatologists according to diagnostic standard for psoriasis were recruited at The Second Affiliated Hospital of Nanhua University (Hengyang, China). All of the enrolled psoriasis patients signed informed consent forms.We collected 70 paired lesional and non-lesional psoriatic skin tissues. 3mm punch biopsies were taken from lesional skin. The non-lesional skin was taken about 5 cm away from the lesion biopsy. All the tissue samples were snap-frozen and stored in liquid nitrogen.

HaCaT, a spontaneously transformed aneuploid immortal keratinocyte cell line, was obtained from the American Type Culture Collection (ATCC, USA), cultured in 10% fetal bovine serum (Gibco, USA) supplemented RPMI-1640 medium (Invitrogen, USA) at 37°C with 5% v/v CO_2_. Primary adult human keratinocytes (HKCs) were purchased from PriCells, China, cultured in keratinocyte-SFM (Gibco, USA) at 37°C with 5% v/v CO_2_. For hypoxia treatment, cells were cultured in hypoxia chamber in a humidified atmosphere with 94% N_2_, 5% CO_2_ and 1% O_2_ for 24h then change to normoxia for additionally indicated time for further assays.

MiR-150 mimics or miR-150 inhibitor (Genepharma, China) was transfected into the indicated target cells at final concentration of 50 nM to achieve miR-150 overexpression or miR-150 inhibition by using Lipofectamine 2000 (Invitrogen). PcDNA3.1/HIF-1α and pcDNA3.1/VEGFA vector at a final concentration of 1 μg/ ml were used to achieve HIF-1α and VEGFA ectopic expression (GeneCopoecia, China).

### Quantitative real time PCR (qPCR) for miRNA and mRNA detection

Trizol reagent (Invitrogen) was used for total RNA extraction following the manufacturer’s instructions. By using miRNA-specific primer, 1 μg total RNA was reverse transcribed using the miScript Reverse Transcription kit (Qiagen, Germany)for miR-150 qRT-PCR. 1 μg RNA was reverse transcribed using the PrimeScript^®^ RT reagent kit with gDNA Eraser (Takara, Japan) for gene mRNA qRT-PCR. cDNA was amplified by qPCR with a 1000-Series Thermal Cyclers real-time PCR detection system (Bio-Rad) and SYBR^®^ Premix Ex Taq™ II (Takara, Japan), according to the manufacturer’s protocol. The expression of mRNA or miRNA was normalized using β-actin and U6 as endogenous control, respectively. All the samples were amplified in triplicate and each experiment was repeated three times. The fold change in expression level was calculated using the 2^−ΔΔCt^ method. Primers for PCR are showed in Table 1.

**Table 1:** Primer sequences for PCR.

Name	Sequence
miR-150	F:5’-TCTCCCAACCCTTGTACCAGT-3’
	R: 5’-GTGCAGGGTCCGAGGT-3’
U6	F:5’- CTCGCTTCGGCAGCACA -3’
	R: 5’- AACGCTTCACGAATTTGCGT -3’
HIF-1α	F: 5’-GAACGTCGAAAAGAAAAGTCTCG-3’
	R:5’- CCTTATCAAGATGCGAACTCACA-3’
VEGFA	F: 5’-CTGTACCTCCACCATGCCAAGT-3’
	R: 5’-AGATGTCCACCAGGGTCTCAAT-3’
β-actin	F: 5’-AGAG GGAAATCGTGCGTGAC-3’
	R: 5’-CAATAGTGATGACCTGGCCGT-3’
VEGFA-3’UTR	F: 5’- AAACTCGAGTGCTAATGTTATTGGTGT-3’
	R: 5’- AAAGCGGCCGCATCCCTGTACCTGTGATC-3’
VEGFA-3’UTR-mutation	F: 5’-ACTGCTGTGGACTTGAGAACTCTAGGGAATGTTCCCACTCA-3’
	R: 5’-TGAGTGGGAACATTCCCTAGAGTTCTCAAGTCCACAGCAGT-3’
HIF1A-3’UTR	F: 5’- AAACTCGAGAAAGCACGTTGAGATATG-3’
	R: 5’- AAAGCGGCCGCCAGAGGCGAAGTTATTTA-3’
HIF1A-3’UTR-mutation	F: 5’- CAGCTATTGAGAATCCTAACTCTAAAGATAATCTTGTTGGA-3’
	R: 5’-TCCAACAAGATTATCTTTAGAGTTAGGATTCTCAATAGCTG-3’

### Western blotting

RIPA buffer (Cell-Signaling Tech., US) was used to homogenize the cells. The expression of HIF-1α and VEGFA in HaCaT cells was detected by performing immunoblotting. Cells were lysed cultured, or transfected in 1% PMSF supplemented RIPA buffer. Protein (30 to 100 μg) were loaded onto SDS-PAGE minigel, and then transferred onto PVDF membrane. The blots were probed with 1:1000 diluted rabbit polyclonal HIF-1α and VEGFA antibody (Abcam, USA) at 4°C overnight, and incubated with HRP-conjugated secondary antibody (1:5000). Signals were visualized using ECL Substrates (Millipore, USA). The protein expression was normalized to endogenous β-actin.

### Luciferase activity

The 3’-UTR of VEGFA gene (position 499–505) and HIF-1α (position 4898–4905) were predicted to be complementary to the sequence of miR-150 according to the online prediction tool, TargetScan. The fragment sequences of wild-type and mutant VEGFA 3’-UTR and HIF-1α 3’-UTR were amplified by PCR with XhoI and NotI site and cloned into the psiCheck-2 vector (Promega, USA), named as VEGFA-wt-3’UTR, VEGFA-mut-3’UTR, HIF-1α-wt-3’UTR, HIF-1α-mut-3’UTR. The primers were showed in Table 1. 293T cells (ATCC, USA) were cultured overnight after being seeded into a 24-well plate, co-transfected with the VEGFA-wt-3’UTR or VEGFA-mut-3’UTR reporter gene plasmid and miR-150 mimics or miR-150 inhibitor; or co-transfected with the HIF-1α-wt-3’UTR or HIF-1α-mut-3’UTR reporter gene plasmid and miR-150 mimics or miR-150 inhibitor. 48 h after transfection, Dual Luciferase Reporter Assay System (Promega, USA) was used to perform the luciferase assays.

### CCK-8 assay

Cell Counting Kit-8 (CCK-8) (Beyotime, Hangzhou, China) was used to measure cell viability. We seeded 0.5×10^4^ cells in each 96-well plate for 24 h, transfected them with the indicated miRNA mimics, inhibitor or pcDNA3.1 vectors, and further incubated cells for 1, 2, 3, 4, 5 days, respectively. At 1 h before the endpoint of incubation we added 10 μl CCK-8 reagents to each well. A microplate reader was used to determine OD_450nm_ value in each well.

### ELISA-BrdU assay

ELISA-BrdU assay was performed to examine the effect of miR-150, HIF-1α or VEGFA, respectively or combined, on cell proliferation. Cells were seeded in 96-well plate at 5×10^3^ cells/well. After 24 h, we removed the medium and transfected cells with the indicated miRNA mimics, miRNA inhibitor, pcDNA3.1/HIF-1α or pcDNA3.1/ VEGFA at 37°C for 24 h. Cell proliferation was detected by using Cell Proliferation ELISA-BrdU Kit (Millipore, USA) following the manufacturer’s protocols.

### Statistical analysis

Data are expressed as the mean ± SD from at least three independent experiments. Statistical analysis was performed using one-way ANOVA or a Newman-Keuls test between groups using Statistical Package for the Social Science (SPSS) (SPSS, Inc., Chicago, IL, USA). *P*<0.05 was considered to indicate a statistically significant difference.

## Results

### MiR-150 is specifically down-regulated in lesional psoriatic skin tissues and associated with clinical characteristics of psoriasis

**Fig 1:**
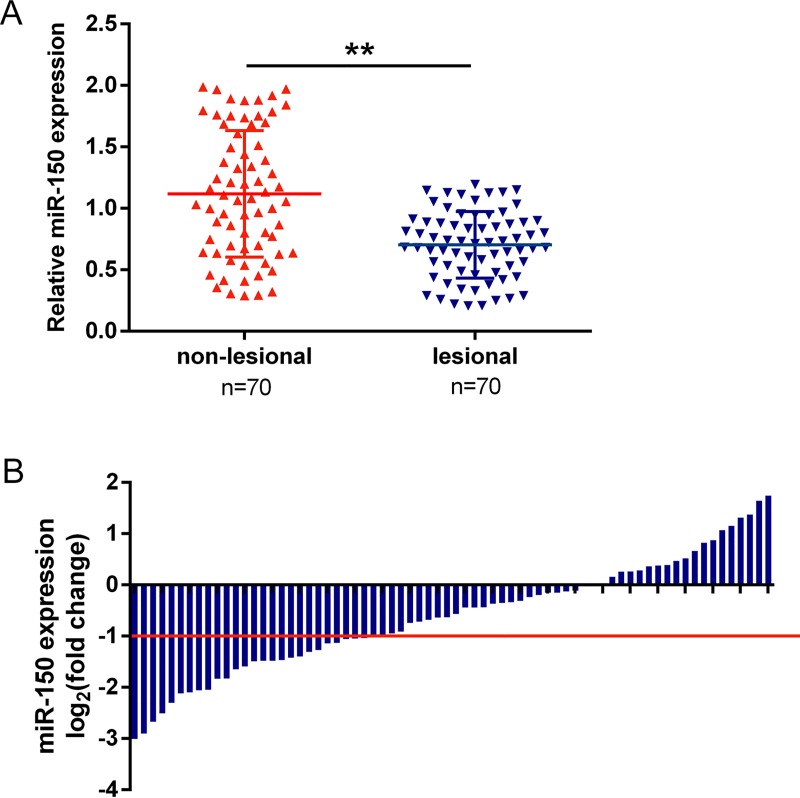
miR-150 is specifically down-regulated in lesional psoriatic skin tissues (A) miR-150 expression was determined in a large panel of 70 paired lesional and non-lesional psoriatic skin tissues using qPCR assays. The data are presented as mean ± SD of three independent experiments. **P<0.001. (B) Expression of miR-150 in 70 pairs of lesional psoriatic skin tissues and their corresponding non-lesional tissues (NLTs) in a training cohort. Expression level of miR-150 was determined by qPCR. Fold change were analyzed using the formula 2^-(ΔΔCT [psoriasis/NLT])^. Red line indicates fold change of miR-150 equal to 2.

QPCR analysis was performed to quantify the expression of miR-150 in lesional and non-lesional psoriatic skin tissues (n = 70). The expression of miR-150 was significantly down-regulated in lesional psoriatic skin tissues, compared with the non-lesional tissues (Fig 1A). To validate the data, we analyzed qPCR results in 70 cases of lesional and non-lesional psoriatic skin tissues in training cohort. Compared with the corresponding non-lesional tissues, miR-150 was significantly down-regulated (more than two-fold [i.e., log_2_ (fold change) > -1]) in 44 of 70 psoriasis cases (62.86%) (Fig 1B). In addition, to correlate miR-150 expression with clinicopathologic features, the 70 psoriasis patients were classified into a relatively high group and a relatively low group using the log_2_ (fold change) = -1 in psoriasis tissues as a cut-off value. Noticeably, lower miR-150 expression in psoriasis was significantly correlated with a longer disease duration (*P* = 0.011), worse Psoriasis Area and Severity Index (PASI) (*P* = 0.019) and larger body surface area (BSA) (*P* = 0.014) (Table 2).

**Table 2:** Correlation between relative miR-150 expression and clinical characteristics of psoriasis.

Characteristics	Mean		miR-150 expression		p
			High	Low	
Age	47.2	<45	22	10	0.17
		> = 45	20	18	
Gender		female	17	6	0.096
		male	25	22	
Disease duration(year)	11.5	<10	25	8	0.011
		> = 10	17	20	
PASI(0–72)^1^	13.4	<10	24	8	0.019
		> = 10	18	20	
BSA(0–100%)^2^	22.6	<20	29	11	0.014
		> = 20	13	17	

^1^ PASI: Psoriasis Area and Severity Index

^2^ BSA: Body Surface Area

### The detailed role of miR-150 in regulating human keratinocytes proliferation

**Fig 2:**
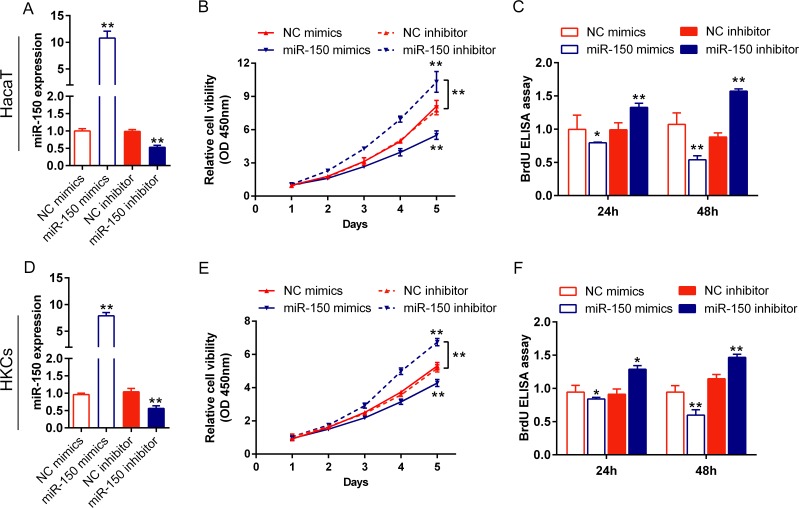
The detailed role of miR-150 in regulating HaCaT cell and HKCs proliferation. NC mimics/miR-150 mimics or NC inhibitor/miR-150 inhibitor was transfected into HaCaT cells (A) and HKCs (D) to achieve miR-150 overexpression or inhibition. The expression efficiency was verified using qPCR assays. The cell viability of HaCaT cells (B) and HKCs (E) transfected with NC mimics/miR-150 mimics or NC inhibitor/miR-150 inhibitor was monitored using CCK-8 assays. The cell proliferation of HaCaT cells (C) and HKCs (F) transfected with NC mimics/miR-150 mimics or NC inhibitor/miR-150 inhibitor was monitored using BrdU-ELISA assays.The data are presented as mean ± SD of three independent experiments. *P<0.05, **P<0.01, vs. NC mimics or NC inhibitor.

As we have indicated the specific low expression of miR-150 in psoriasis tissues and its correlation with clinical characteristics of psoriasis, we further detected the detailed role of miR-150 in HaCaT cell and HKCs proliferation. Cells were transfected with NC mimics/miR-150 mimics or NC inhibitor/miR-150 inhibitor, and the transfection efficiency was verified using qPCR assay (Fig 2A and 2D). Then viability of HaCaT and HKCs (by CCK-8 assay) and proliferation (by BrdU-ELISA assay) was monitored in response to miR-150 overexpression and inhibition. As showed in Fig 2B and 2C, HaCaT cells’ viability and proliferation was significantly inhibited by miR-150 overexpression, while promoted by miR-150 inhibition, compared with negative control (NC) group. Similarly, HKCs’ viability and proliferation was also significantly inhibited by miR-150 overexpression, while promoted by miR-150 inhibition, compared with NC group (Fig 2D and 2E). These data indicated that miR-150 inhibits human keratinocytes viability and proliferation.

### Effects of miR-150 on human keratinocytes and expression of HIF-1α and VEGFA in hypoxic condition

**Fig 3:**
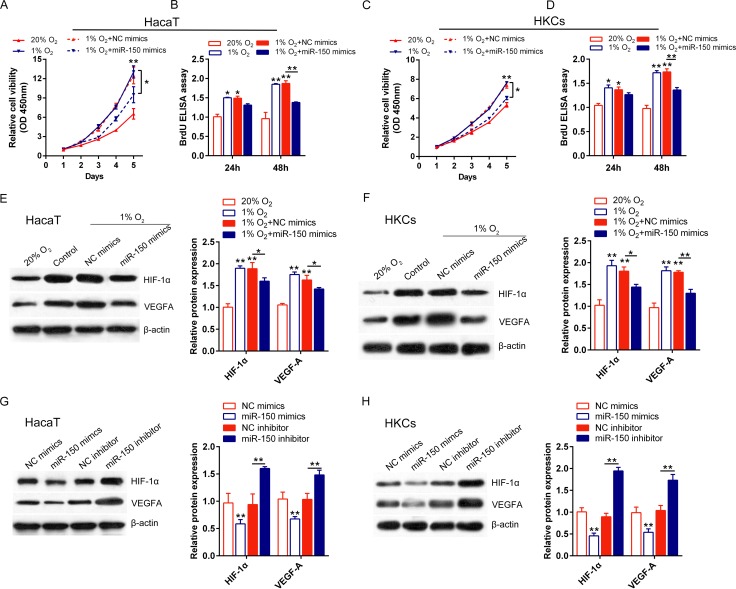
Effects of miR-150 on human keratinocytes and expression of HIF-1α and VEGFA in hypoxic condition The cell viability of HaCaT cells (A) and HKCs (C) transfected with NC mimics/miR-150 mimics in 1% O2 condition or 20% O2 condition was monitored using CCK-8 assays. The cell proliferation of HaCaT cells (B) and HKCs (D) transfected with NC mimics/miR-150 mimics in 1% O2 condition or 20% O2 condition was monitored using BrdU-ELISA assays. (E) and (F) The protein expression of HIF-1α and VEGFA in HaCaT cells and HKCs transfected with NC mimics/miR-150 mimics in 1% O2 condition or 20% O2 condition was monitored using Western blot assays. (G) and (H) The protein expression of HIF-1α and VEGFA in HaCaT cells and HKCs transfected with NC mimics/miR-150 mimics or NC inhibitor/miR-150 inhibitor was monitored using Western blot assays. The data are presented as mean ± SD of three independent experiments. *P<0.05, **P<0.01.

As reported by previous studies, during the psoriasis course, the excessive proliferation of keratinocytes causes a hypoxic micro environment, and subsequent accelerated angiogenesis. To validate the effects of miR-150 on HaCaT cells and HKCs in hypoxic condition, functional assays were generated in 1% O_2_ condition and 20% O_2_ condition, respectively. HaCaT cells and HKCs were divided into four groups: 1% O_2_ condition, 20% O_2_ condition, 1% O_2_ + NC mimics, 1% O_2_ + miR-150 mimics. The cell viability and proliferation of HaCaT cells and HKCs were monitored by CCK-8 and BrdU-ELISA assays. Results showed that the cell viability and proliferation of HaCaT cells and HKCs were significantly down-regulated in 20% O_2_ condition while promoted in 1% O_2_ condition; the hypoxic condition-induced cell proliferation could be partially restored by miR-150 overexpression (Fig 3A–3D). Next, the protein expression of HIF-1α and VEGFA was monitored in hypoxic or normal condition by using Western blot assays. The protein expression of HIF-1α and VEGFA was determined in the indicated four groups. Results showed that both protein expression of HIF-1α and VEGFA in HaCaT cells and HKCs were down-regulated in 20% O_2_ condition while promoted in 1% O_2_ condition; the hypoxic condition-induced HIF-1α and VEGFA protein expression could be partially restored by miR-150 overexpression (Fig 3E and 3F). Then we further validate whether miR-150 had the capability to regulate HIF-1α and VEGFA protein expression in HaCaT cells and HKCs. Results from Western blot assays showed that both protein expression of HIF-1α and VEGFA were down-regulated by miR-150 overexpression while promoted by miR-150 inhibition; the suppression of HIF-1α and VEGFA protein expression mediated by miR-150 mimics could be partially restored by miR-150 inhibitor (Fig 3G and 3H). These data indicated that miR-150 could regulate HIF-1α and VEGFA protein expression, and subsequently modulate human keratinocytes viability and proliferation in hypoxic condition.

### MiR-150 regulates HIF-1α and VEGFA by direct targeting

**Fig 4:**
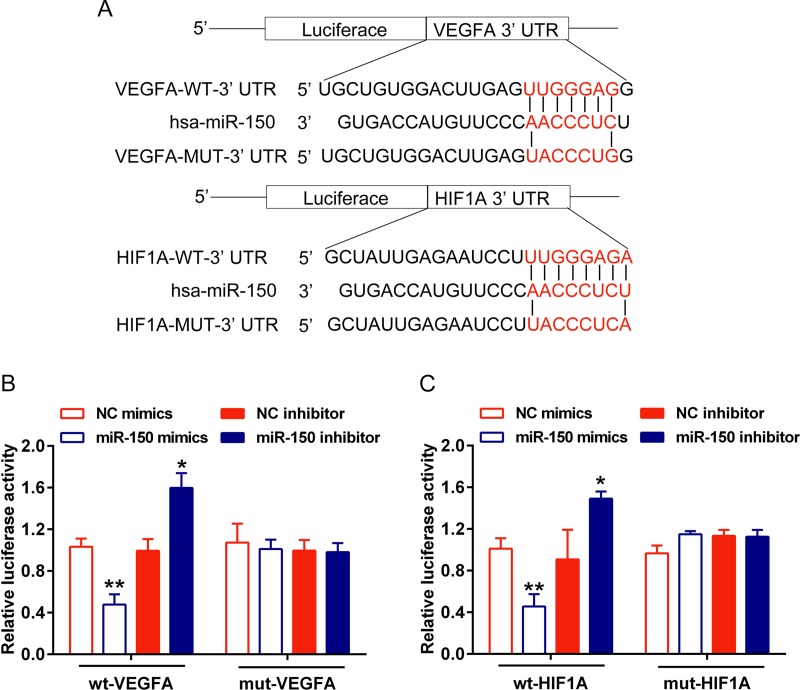
miR-150 regulates HIF-1α and VEGFA by direct targeting (A) A wt-HIF-1α 3’UTR luciferase reporter vector (wt-HIF-1α), a wt-VEGFA 3’UTR luciferase reporter vector (wt-VEGFA), a mut-HIF-1α 3’UTR luciferase reporter vector (mut-HIF-1α) and a mut-VEGFA 3’UTR luciferase reporter vector (mut-VEGFA) with mutations on miR-150 binding sites of the 3’UTR of HIF-1α or VEGFA was constructed. (B, C) The indicated luciferase reporter gene vectors were co-transfected into HaCaT cells with NC mimics/miR-150 mimics or NC inhibitor/miR-150 inhibitor, and the luciferase activity was monitored in each group using dual luciferase assays. The data are presented as mean ± SD of three independent experiments. *P<0.05, **P<0.01.

We have demonstrated that miR-150 inhibits HIF-1α and VEGFA protein expression. Next we further confirmed the regulation of HIF-1α and VEGFA by miR-150 and the underlying mechanism. To identify the functional mechanism of miR-150 on HIF-1α (position 4898–4905) and VEGFA (position 499–505), with the help of bioinformatics search (Diana, microinspector and targetscan, etc.), we found a putative binding sites for miR-150 in the 3’UTR coding region of HIF-1α and VEGFA, respectively (Fig 4A). Accordingly, HIF-1α and VEGFA luciferase reporter vectors were constructed, named wt-HIF-1α, mut-HIF-1α, wt-VEGFA and mut-VEGFA, by cloning those sequences into the psiCheck-2 vector. The mut-HIF-1α and mut-VEGFA harbors the predicted mutated sites. Co-transfection with NC mimics/miR-150 mimics or NC inhibitor/miR-150 inhibitor and the above reporter vectors showed that for the wild type, the luciferase activity was largely abolished by introduction of miR-150; once the predicted sites were mutated, this effect was gone (Fig 4B and 4C). These data investigated that miR-150 inhibited HIF-1α and VEGFA by directly binding to their 3’UTR, respectively.

### The role of miR-150/HIF-1α and miR-150/VEGFA in regulating human keratinocytes proliferation

**Fig 5:**
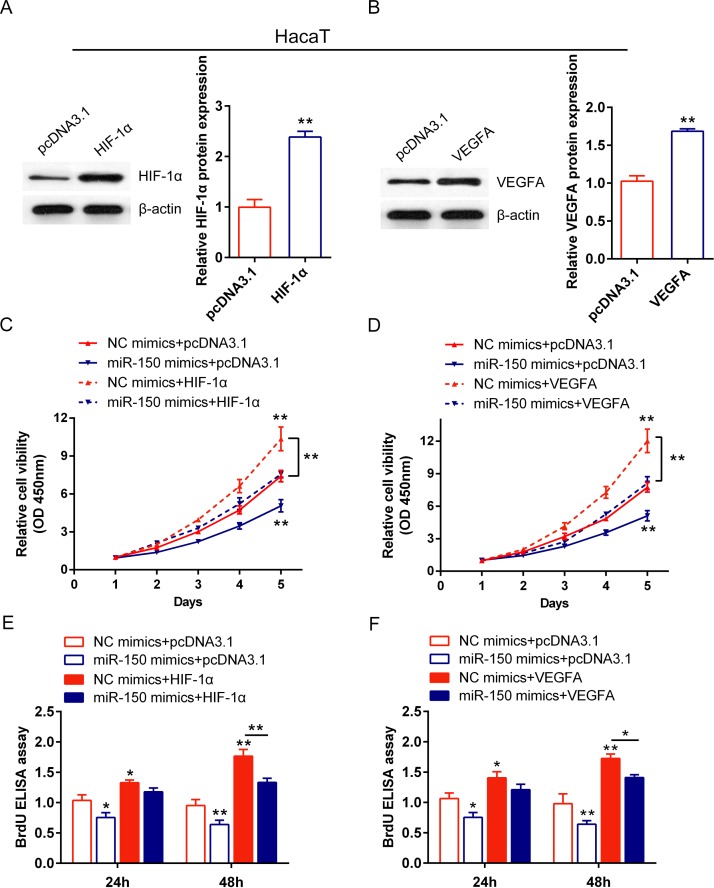
The role of miR-150/HIF-1α and miR-150/VEGFA in regulating HaCaT cell proliferation (A, B) pcDNA3.1/HIF-1α and pcDNA3.1/VEGFA was transfected into HaCaT cells to achieve ectopic expression of HIF-1α or VEGFA, respectively. The protein expression of HIF-1α and VEGFA was verified using Western blot assays. (C) HaCaT cells were co-transfected with pcDNA3.1/HIF-1α and NC mimics/miR-150 mimics, and the cell viability was monitored in each group using CCK-8 assays. (D) HaCaT cells were co-transfected with pcDNA3.1/VEGFA and NC mimics/miR-150 mimics, and the cell viability was monitored in each group using CCK-8 assays. (E) HaCaT cells were co-transfected with pcDNA3.1/HIF-1α and NC mimics/miR-150 mimics, and the cell proliferation was monitored in each group using BrdU-ELISA assays. (D) HaCaT cells were co-transfected with pcDNA3.1/VEGFA and NC mimics/miR-150 mimics, and the cell proliferation was monitored in each group using BrdU-ELISA assays. The data are presented as mean ± SD of three independent experiments. *P<0.05, **P<0.01.

**Fig 6:**
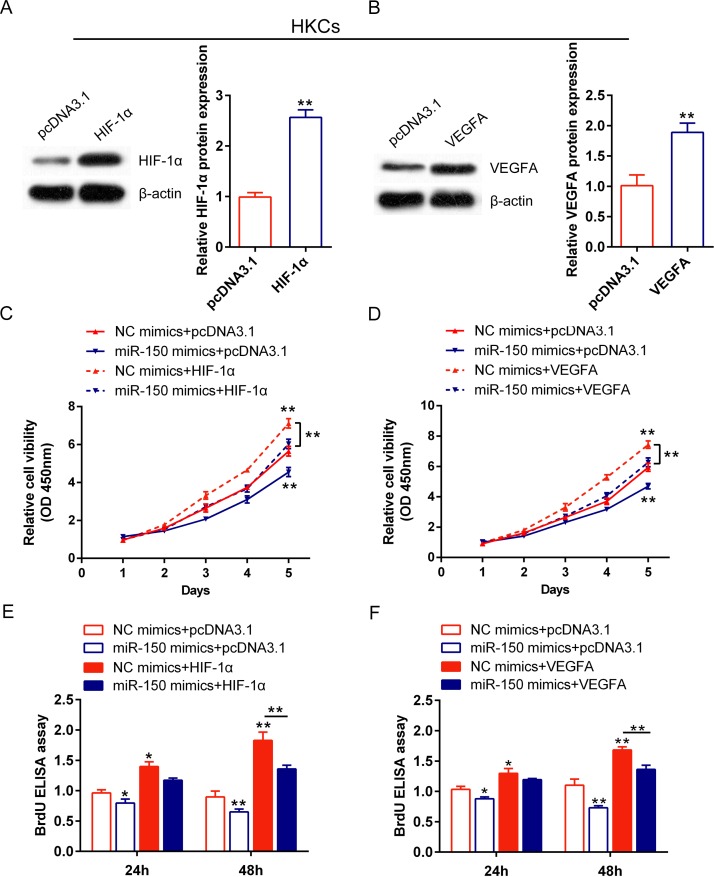
The role of miR-150/HIF-1α and miR-150/VEGFA in regulating HKCs proliferation (A, B) pcDNA3.1/HIF-1α and pcDNA3.1/VEGFA was transfected into HKCs to achieve ectopic expression of HIF-1α or VEGFA, respectively. The protein expression of HIF-1α and VEGFA was verified using Western blot assays. (C) HaCaT cells were co-transfected with pcDNA3.1/HIF-1α and NC mimics/miR-150 mimics, and the cell viability was monitored in each group using CCK-8 assays. (D) HKCs were co-transfected with pcDNA3.1/VEGFA and NC mimics/miR-150 mimics, and the cell viability was monitored in each group using CCK-8 assays. (E) HKCs were co-transfected with pcDNA3.1/HIF-1α and NC mimics/miR-150 mimics, and the cell proliferation was monitored in each group using BrdU-ELISA assays. (D) HKCs were co-transfected with pcDNA3.1/VEGFA and NC mimics/miR-150 mimics, and the cell proliferation was monitored in each group using BrdU-ELISA assays. The data are presented as mean ± SD of three independent experiments. *P<0.05, **P<0.01.

We have investigated that miR-150 inhibits human keratinocytes proliferation, and that miR-150 inhibits HIF-1α and VEGFA expression by direct binding to their 3’UTR, respectively. To validate whether miR-150 regulated HIF-1α and VEGFA to modulate keratinocytes proliferation, a series functional analysis was generated. pcDNA3.1/HIF-1α and pcDNA3.1/VEGFA was transfected into HaCaT cells and HKCs, respectively, to achieve HIF-1α and VEGFA ectopic expression. The expression efficiency of HIF-1α and VEGFA was verified using Western blot assays (Figs 5A, 5B, 6A and 6B). Then the HaCaT cells and HKCs were divided into eight groups. For miR-150/HIF-1α: NC mimics + pcDNA3.1, NC mimics + pcDNA3.1/HIF-1α, miR-150 mimics + pcDNA3.1, miR-150 mimics + pcDNA3.1/HIF-1α; for miR-150/VEGFA: NC mimics + pcDNA3.1, NC mimics + pcDNA3.1/VEGFA, miR-150 mimics + pcDNA3.1, miR-150 mimics + pcDNA3.1/VEGFA. The cell viability (by CCK-8 assays) and proliferation (by BrdU-ELISA assays) was monitored in each group. Results from CCK-8 assays showed that the cell viability of HaCaT and HKCs was significantly down-regulated by miR-150 overexpression while up-regulated by either HIF-1α or VEGFA ectopic expression; the inhibitory effect of miR-150 on cells’ viability could be partially restored by either HIF-1α or VEGFA ectopic expression (Figs 5C, 5D, 6C and 6D). Similar results were observed with BrdU-ELISA assays: the cell proliferation of HaCaT and HKC2 was significantly down-regulated by miR-150 overexpression while up-regulated by either HIF-1α or VEGFA ectopic expression; the inhibitory effect of miR-150 on cells’ proliferation could be partially restored by either HIF-1α or VEGFA ectopic expression (Figs 5E, 5F, 6E and 6F). These data indicated that miR-150 could affect human keratinocytes proliferation via HIF-1α and VEGFA.

### Expression of HIF-1α and VEGFA and their correlation with miR-150 in psoriatic skin

**Fig 7:**
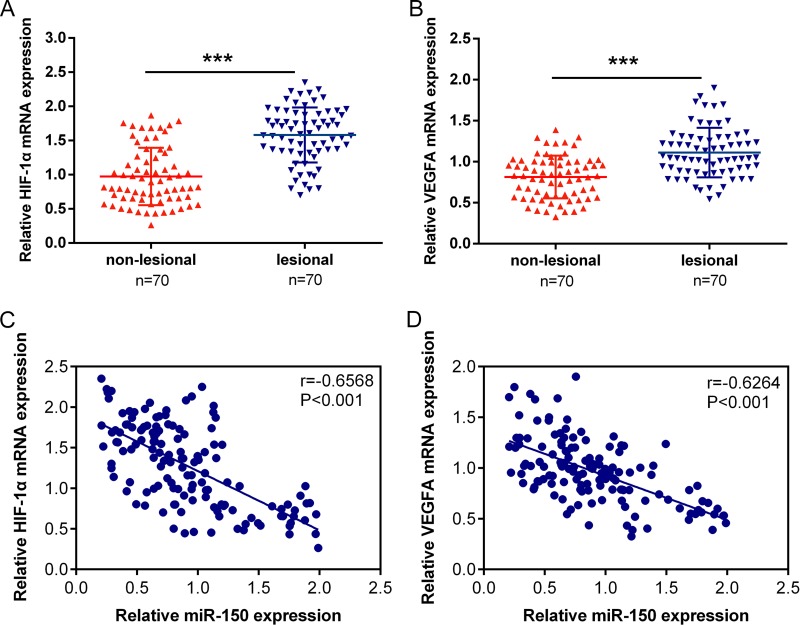
Expression of HIF-1α and VEGFA and their correlation with miR-150 in psoriasis lesional tissues and non-lesional tissues (A, B) The expression of HIF-1α and VEGFA was monitored in a large panel of 70 paired lesional and non-lesional psoriatic skin tissues using qPCR assays. The data are presented as mean ± SD of three independent experiments. ***P<0.005. (C, D) The correlation between miR-150 and HIF-1α, between miR-150 and VEGFA was analyzed using Spearman’s correlation analysis.

After detecting the functional roles of miR-150, HIF-1α and VEGFA in HaCaT cells and HKCs, we determined their expression levels of HIF-1α and VEGFA and the correlation with miR-150 in lesional amd non-lesional psoriatic skin tissues. Results from qPCR assays showed that both HIF-1α and VEGFA mRNA was up-regulated in psoriasis tissues (Fig 7A and 7B). By using Spearman’s correlation analysis, we observed an inverse correlation between miR-150 and HIF-1α, an inverse correlation between miR-150 and VEGFA in psoriatic skin (Fig 7C and 7D).

## Discussion

During the psoriasis process, proliferating keratinocytes exhibit hypoxia-induced VEGFA expression, which initiates a process that aims to achieve the proper flow of blood through the lesional skin [28, 29]. However, changes in the miRNA profile that upregulated VEGFA expression level upon hypoxia is still unclear. The present study aimed to investigate the functions of miRNAs targeting VEGFA and hypoxia related factor HIF-1α. Initially, we examined the expression of miR-150 which was reported to be specifically down-regulated expressed in psoriatic skins [20], and performed a series functional analysis to figure out the detailed role of miR-150 in keratinocytes, in normal or hypoxia condition. Additionally, we observed the effect of miR-150 knockdown or overexpression on HIF-1α and VEGFA protein expression in keratinocytes in normal or hypoxia condition. Finally, we demonstrated the regulation of HIF-1α and VEGFA by miR-150 via direct binding to HIF-1α and VEGFA’s 3’UTR, and the effect of miR-150/HIF-1α and miR-150/VEGFA on keratinocytes’ proliferation.

MicroRNAs (miRNAs) represent an abundant class of small, evolutionarily conserved, non-coding RNA molecules that post-transcriptionally regulate gene expression. These non-coding RNAs are fundamental to human life and disease states [30]. In psoriasis, miRNA expression disorders have been reported [31, 32]. To date, more than 250 miRNAs have been reported as aberrantly expressed in psoriasis tissue, the majority of which are found in peripheral blood or involved psoriatic skin, but only small subsets of these dysregulated miRNAs in psoriasis have confirmed mRNA targets with established biological functions in the skin [19]. In the present study, we determined the expression level and detailed function of miR-150, which was reported to be down-regulated in psoriasis tissues [20]. Consistent with the indicated study, a down-regulated expression of miR-150 was observed, and we found that the lower miR-150 expression was associated with longer disease duration, worse PASI and larger BSA in psoriasis patients. In addition, miR-150 overexpression inhibited HaCaT cells and HKCs’ proliferation. However, the mechanism by which miR-150 inhibits the cell proliferation of keratinocytes still remains to be validated.

Basically, miRNAs exert their functions through binding to the 3’UTR of target mRNA genes and subsequently disrupts translation or triggers mRNA degradation [19]. Excessive proliferation of keratinocytes in psoriasis causes local environmental hypoxia, leading to increased expression of HIF-1α [12]; microenvironment hypoxia accelerates vascular growth to supply the oxygen required for cell growth, along with the elevated expression of VEGFA during this process [33]. Besides, HIF-1α and VEGFA were reported as crucial regulators during the pathogenesis of psoriasis [33, 34]. As we demonstrated that miR-150 inhibits HaCaT cells and HKCs’ proliferation, here we performed functional and mechanistic analysis to validate whether miR-150 regulates keratinocytes’ proliferation through regulating HIF-1α and VEGFA in hypoxia condition. Results showed that, in hypoxia condition (1% O_2_), the cell viability and proliferation were significantly promoted, along with a significantly increased expression of HIF-1α and VEGFA, compared with in normal condition. However, miR-150 overexpression repressed the cell proliferation and the expression of HIF-1α and VEGFA. The promotive effect of hypoxia on’ cell proliferation and HIF-1α and VEGFA expression could be partially restored by miR-150. These indicated that in psoriasis cells, miR-150 inhibits the cell proliferation induced by hypoxia, as well as the accelerated expression of HIF-1α and VEGFA.

As we mentioned, miRNAs commonly exert their regulatory function through binding to the 3’UTR of their target mRNA genes. Recent studies reported that miR-150 could regulate HIF-1α and VEGFA by targeting in many kinds of cancers [27, 35]. We indicated that miR-150 inhibits HIF-1α and VEGFA expression in HaCaT cells and HKCs to regulate cell proliferation. To confirm the mechanism by which miR-150 inhibits HIF-1α and VEGFA expression, we generated luciferase analysis. As expected, miR-150 could directly bind to the predicted binding site on the 3’UTR of HIF-1α and VEGFA, respectively. These data indicated the regulation of HIF-1α and VEGFA by miR-150 through miR-150 direct binding to their 3’UTR, respectively.

Having clarified that miR-150 regulates HIF-1α and VEGFA expression to modulate the proliferation of HaCaT cell and HKCs; we further validate the combined effect of miR-150 with HIF-1α or VEGFA on HaCaT cells and HKCs proliferation. Results showed that miR-150 effectively inhibits cell proliferation; both HIF-1α and VEGFA could promote cell proliferation, while this promotive effect could be partially restored by miR-150 overexpression. Finally, we validated that the expression of HIF-1α and VEGFA are up-regulated in lesional psoriatic skin tissues compared to non-lesion tissues. The expression of HIF-1α and VEGFA are both inversely correlated with miR-150, respectively. These data indicated that miR-150-dependent HIF-1α and VEGFA regulation could be a target to control psoriasis considering that these factors are involved in angiogenesis and proliferation of keratinocytes.

Taken together, we demonstrate that the expression of miR-150 is significantly down-regulated in lesional psoriatic skin, and is specifically related to HIF-1α and VEGFA during the psoriasis process. Besides, miR-150 regulates HIF-1α and VEGFA by direct binding to the 3’UTR of HIF-1α and VEGFA to inhibit human keratinocytes’ proliferation, suggesting that miR-150, HIF-1α and VEGFA may serve as useful diagnostic markers and novel targets for treatment strategies of psoriasis.

## Supporting information

S1 File Western blot scanning images. (RAR)

S2 File Figs 1–7 data. (XLSX)
